# Primary signet ring cell carcinoma of the cervix: case report and literature review

**DOI:** 10.3332/ecancer.2024.1671

**Published:** 2024-02-16

**Authors:** Milagros Abad-Licham, Christian Cotrina, Andric Guerrero, Katherine Gómez, Juan Astigueta

**Affiliations:** 1 Faculty of Medicine, Universidad Privada Antenor Orrego, Trujillo 13008, Perú; 2 Department of Oncological Pathology, Instituto Regional de Enfermedades Neoplásicas, Trujillo 13007, Peru; 3 Centre of Excellence in Oncological Pathology, Trujillo, Peru; 4 Department of Oncology Surgery, Instituto Regional de Enfermedades Neoplásicas, Trujillo 13007, Peru; a https://orcid.org/0000-0002-3530-6937; b https://orcid.org/0009-0006-8767-7314; c https://orcid.org/0000-0002-2619-1920; d https://orcid.org/0000-0002-9097-0224; e https://orcid.org/0000-0001-5984-3270

**Keywords:** cervix, carcinoma, signet ring cell

## Abstract

**Objective:**

To report an infrequent case of primary signet ring cell carcinoma of the cervix (PSRCC), review the literature and evaluate the clinicopathological characteristics.

**Material and methods:**

A 51-year-old female patient, with 3 years of disease characterised by gynaecologic bleeding and pelvic pain. On examination, cervix replaced by tumour and infiltrated parametria; with cytology and histology of adenocarcinoma with cells in a signet ring pattern. Disease extension studies were negative. Classified as PSRCC stage IIIB, chemotherapy and radiotherapy were indicated, but the patient died a month later. The bibliographic search included publications up to July 2023.

**Results:**

32 cases with a mean age of 47.6 years were identified. The most frequent symptoms were uterine bleeding and abdominal and pelvic pain. The initial stages were treated surgically, some with adjuvant chemotherapy and/or radiotherapy, with favourable responses; a difference from advanced cases. The average survival was 17.6 months.

**Conclusion:**

PSRCC is rare, with 32 cases reported according to the review. The expression with immunohistochemical and molecular techniques for the human papilloma virus can help confirm the gynaecological primary, but its diagnosis is one of exclusion, since its morphological pattern is related to other primaries, mainly digestive.

## Introduction

Cervical cancer is a public health problem, with an estimated incidence of more than 600,000 new cases in 2020 and which, according to the World Health Organisation, represents the fourth most common cancer worldwide [1], whose pathogenesis, in most cases, is linked to human papilloma virus (HPV), infection that is distributed unevenly, mainly affecting patients from low and middle income countries, including Peru [1–3].

Two main histological types have been described: squamous cell carcinoma (SCC) accounting for approximately 70%–75% of cases and adenocarcinoma (AC) with 10%–25%, the latter with an increasing incidence in recent decades [2]. In both neoplasms, HPV is the most important risk factor in its pathogenesis, with serotype 16 being the most related to SCC and 18 to AC, which could explain the difference in their clinical behaviour and response to treatment [2–5].

Although SCC is the most common and most studied histological type, AC is presented as an interesting heterogenous group of malignant tumours that represent between 10% and 25% of all cervical carcinomas, and that based on cellular architecture and morphology, they can be divided into two subtypes: usual (endocervical) and mucinous [2, 3]; the latter represents 15% of cervical ACs and are characterised by having more than 50% mucin occupying the intracytoplasmic space [4–7]. In turn, mucinous AC is subdivided into the intestinal, signet ring cell, minimal deviation and villoglandular variants, each with different morphology, biological and clinical behaviour [4, 5].

PSRCC is an extremely rare presentation variant, being first published in 1990 by Moll [6, 7]. Its morphology is typical and shows cells with an eccentric nucleus due to the presence of large intracytoplasmic vacuoles, morphological characteristics of gastric malignant neoplasms [5, 6], reason for which its presence in the cervix allows us to propose the diagnosis of metastatic disease, which can also originate in the colon, breast, gallbladder, lungs, caecal appendix, bladder or ovaries [4, 6–9]. Its biological behaviour places it as a poorly differentiated, very aggressive carcinoma with a poor prognosis [5, 7], whose pathogenesis is still uncertain; however, it is believed that the ErbB2/ErbB3 pathway plays an important role [5].

In relation to HPV infection, it is known that it is present in 85%–90% of cervical ACs, with serotype 18 being the most identified in the different series [5, 10, 11]. Stolnicu *et al* [12–14] collected 409 cases of ACs, from 7 different institutions, who underwent immunohistochemistry for p16, p53, vimentin and progesterone receptor, in addition to chromogenic *in situ* hybridisation to high-risk HPV, finding that the usual type (73%) and the mucinous type (9%) with its intestinal variants, signet ring, unspecified mucinous and stratified mucin-producing carcinoma, were positive for the tests that demonstrated their association with the virus. In that sense, the presence of HPV in signet ring cell carcinoma could support the primary cervical origin [12–14].

Given the histological finding of a signet ring cell carcinoma in the uterine cervix, it is necessary to know the patient’s oncological history, analyse the clinical presentation, the physical examination, the results of the imaging studies, the endoscopic findings to conclude by exclusion that it is a PSRCC [15–17].

Due to the rarity of the disease, the treatment is not defined, so there are no established guidelines or management protocols. This includes surgery, chemotherapy and radiotherapy, as monotherapy or in combination [18–20].

We present a case of this rare entity, the first reported in our country; additionally, we discuss the management and development of the disease based on the results of the literature review.

## Case report

51-year-old female patient, native of Venezuela, without personal or family oncological history, with obstetric formula G4 P4004. Admission to the gynaecology service of the Northern Regional Institute of Neoplastic Diseases (Peru), with a duration of illness of 3 years, characterised by intermittent vaginal bleeding and pelvic pain of moderate intensity. On physical examination, the patient was thin, pale, in whose soft and depressible abdomen, a tumour was palpated in the hypogastrium. In the gynaecological evaluation, the cervix was replaced by a 7 cm crateriform tumour with abundant necrotic tissue, easily bleeding, affecting up to the middle third of the vagina, on rectal examination, the parameters were found infiltrated and fixed to the pelvic bone. Peripheral lymphadenopathies were not palpated.

**Figure 1.**
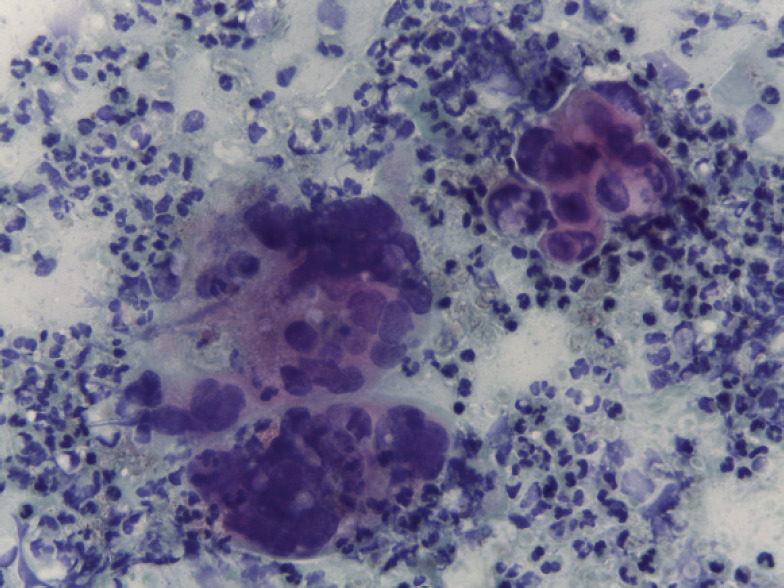
Cervical-vaginal cytology, AC cells with eccentric hyperchromatic nuclei and cytoplasmic vacuolation are observed on an inflammatory background (20× PAP).

**Figure 2.**
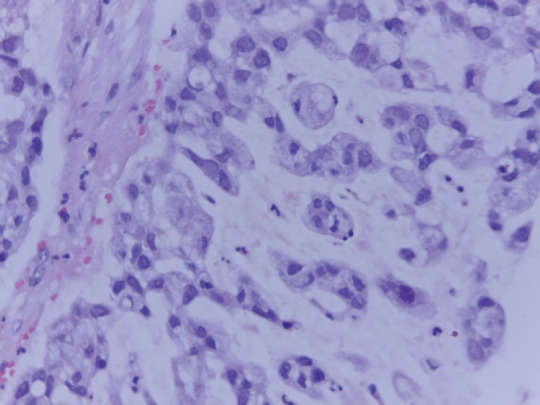
Histological section of cervical tumour in which AC cells are seen on a background of mucin in a signet ring pattern with eccentric nucleus and vacuolated cytoplasm (20× HE).

**Figure 3.**
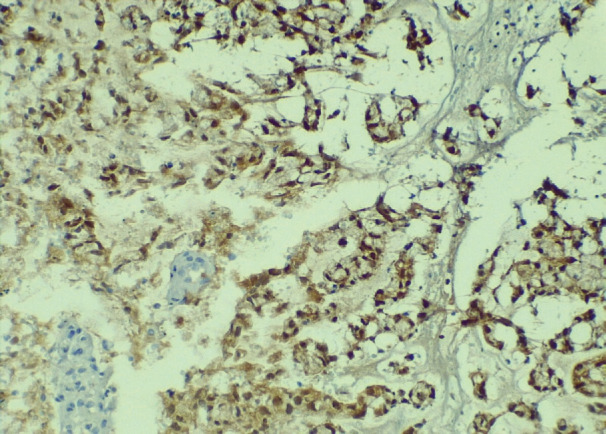
Positive nuclear staining for p16 is observed in signet ring pattern AC cells (10× IHQ).

A cervical-vaginal smear was performed, with a cytological diagnosis of poorly differentiated AC with abundant distribution of signet ring cells in the cellular debris and inflammatory component. The cells presented with a hypochromatic, eccentric nucleus and abundant vacuolated cytoplasm (Figure 1), and histology from a biopsy of the cervical tumour showed signet ring cell carcinoma. In the histological section, a malignant neoplasm comprising groups of AC cells in a signet ring pattern with eccentric nuclei and vacuolated cytoplasm was observed on a mucin background (Figure 2). A p16 immunohistochemical study showed positive diffuse and intense expression in comparison to HPV (Figure 3).

Thoracic, abdominal and pelvic tomography showed an extensive tumour measuring 154 × 102 mm replacing the cervix and compressing the sigmoid rectum and bladder; no pelvic or retroperitoneal lymphadenopathy; no visceral abdominal or pulmonary metastases.

The clinical evaluation was completed with upper and lower gastrointestinal endoscopy, tumour markers and mammography, all of which were negative for malignant neoplasm. With a diagnosis of stage IIB PSRCC, the patient was scheduled for chemotherapy and radiotherapy, but did not return to the institution, and died within a month following disease progression.

## Materials and methods

The medical literature review was conducted using PubMed, EBSCO and Google Scholar databases, with the MeSH terms: ‘carcinoma’, ‘neoplasm’, ‘signet ring cell’, ‘uterine cervical’ and ‘cancer’. The search included publications in English and Spanish, without specifying dates, up to July 2023. Information regarding country, year, number of cases, symptoms, anatomopathological studies, clinical studies, treatment type, follow-up period, survival and disease stage at the time of publication was searched. The information was organised using a data collection tool (Excel) and analysed descriptively.

### Ethical aspects

For the publication of this case, considering the importance of contributing new data for an uncommon disease, authorisation from the Institutional Ethics in Research Committee of the institution was requested and obtained. Pertinent precautions were taken to ensure the confidentiality of the information, such as the anonymity of the patient. The images shown are from the anatomopathological study, without any reference, in order not to violate privacy.

## Discussion

In the initial bibliographic search, 29 publications on PSRCC were identified, with 1 rejected for review purposes as it only evaluated the anatomopathological study. All were reports on one or two cases; ultimately, a total of 32 patients were included (Table 1).

The first case report was from 1990 in the United States; later, others from different countries were added: 12 from America, 11 from Asia, 7 from Europe and 2 from Africa. In South America, there is only a report of one case in Colombia in 2022 [11], and ours, which is the first in Peru.

In the review, the average age at diagnosis was 47.6 years, with a range from 29 to 80; the age bracket in which the patient reported here was found. The most frequent symptoms were abnormal blood in the urine, as in our case, followed by post-coital bleeding, post-menopausal (bleeding) and abdominopelvic pain. Less frequently, they presented with thromboembolism, lymphadenopathy or vaginal discharge.

Only 14 cases included information from the cytological study of the cervix: 4 were diagnosed as poorly differentiated AC [21–24], as in our case; 2 as squamous intraepithelial lesion [11, 16]; 4 as atypical cytology [8, 18, 19, 25]; 3 as negative for malignant neoplasm [9, 15, 26]; and 1 inadequate sample [27]. In the cases of AC, signet ring cells, hyperchromatic nucleus displaced in the periphery and large cytoplasm with large mucin vacuoles were identified; characteristics that initially suggest, by frequency, an extra-cervical origin [22].

The presence of HPV in signet ring cell carcinoma may support a primary cervical origin, as in the case we report. It is important to mention that the immunohistochemical test for p16 is an indirect marker of infection for this virus. Studies performed by Stolnicu *et al* [12–14] showed that grouping based on association with HPV allows for differentiation in the clinical behaviour of the neoplasm. The authors note that tumours related to the virus are smaller and less aggressive, with higher overall survival and disease-free survival in comparison with its non-HPV associated counterpart [12–14]. The specific case of PSRCC, HPV 18 is the most frequently identified genotype [4, 7, 10].

According to the review, patients’ average survival was 17.6 months. 10 died with evidence of disease; 17 were alive with no evidence of same; 2 were alive with clinical evidence; and in 3 cases, this information was not recorded. The shortest survival time was 1 month, described by Lazhar *et al* [4], in a patient in stage IV, and the longest was 120 months, reported by Lowery *et al* [27], in a case at stage IB1 treated with radiotherapy and surgery. As shown in Table 1, it was concluded that 44% of cases were diagnosed at stages III and IV; in the reported case, due to the involvement of the parametrium and extension to the pelvic wall, it was classified as stage IIIB.

Due to the small number of patients, there is no defined management protocol for this disease; some authors recommend treating it as conventional AC of the cervix [10], and others, such as Salmen *et al* [15], suggest that, in unresectable cases, chemo-radiation with brachytherapy following hysterectomy is one option to consider. Our patient, for extra-medical reasons, disappeared from view and did not receive any treatment.

From the existing data, it appears that the survival rate was significantly higher in patients in clinical stage I treated surgically, with or without chemotherapy and/or radiotherapy [6, 7, 27]; the opposite occurs with the disease in advanced stages with aggressive behaviour [4, 5, 28–31].

Finally, before the presence of the signet ring cell carcinoma of the cervix, it is important to define the origin of the neoplasm, considering that it is more common in the stomach, colon, mammary gland, caecal appendix, bladder and ovary [6, 8, 15]. In this sense, complementary clinical evaluations are mandatory as was done with our patient whose laboratory, imaging and endoscopic studies were negative for another primary.

## Conclusion

PSRCC is very rare, with only 32 cases reported according to the literature review. Its diagnosis is one of exclusion, after highlighting other possible primaries. Expression with immunohistochemistry and molecular techniques for HPV can help to confirm the gynaecological origin. In the early stages, long survivals are reported with surgery as the cornerstone of treatment with or without adjuvant chemotherapy and/or radiotherapy, but with advanced disease, the prognosis is fatal in the short term.

## Conflicts of interest

None of the authors declare any conflicts of interest.

## Funding

Self-financed work.

## Author contribution

Milagros Abad-Licham: preparation of the document from its conception and design to the acquisition of the information, review of intellectual content, preparation of photos, participation in graphic designs and approval of the version sent to the editorial process.

Christian Cotrina: review of intellectual content, data collection, preparation of tables and approval of the version sent to the editorial process.

Andric Guerrero: bibliographic review, intellectual content and approval of the version sent to the editorial process.

Katherine Gómez: bibliographic review, intellectual content and approval of the version sent to the editorial process.

Juan Astigueta: preparation of the document from its conception and layout to the acquisition of the information, review of intellectual content, preparation of photos, tables and participation in layout of the graphic material and approval of the version sent to the editorial process.

## Figures and Tables

**Table 1.** Clinicopathological details of reported cases of PSRCC, including the current one.

Author	Author	Age	Symptoms	Cervical cytology	Stage	Treatment	Current status
1	Moll (USA 1990)	50	SPC	NR	III	C + RT	FCEE-10M
2	Mayorga (España 1997)	68	SPM	NR	IB2	QT + C	VSEE-35M
3		74	SPM	NR	IB2	C	VSEE-25M
4	Haswani *et al* [28] (Canadá 1998)	33	ASINT	NR	IIIB	RT + QT	FCEE-10M
5		38	SPC	NR	IB1	C + RT	VSEE-18M
6	Cardosi *et al* [30] (USA 1999)	53	SPM	NR	NR	C + QTRT	VSEE-6M
7	Moritani *et al* [25] (USA 2004)	29	SUA	Atypical	IIIC1	C + QT	VSEE-6M
8	Suárez-Peñaranda *et al* [29] (España 2007)	80	FV	NR	IIIB	QT + QTRT	FCEE-19M
9	Insabato (Italia 2007)	46	SUA	NR	IB1	C	VSEE-36M
10	Veras *et al* [26] (USA 2009)	36	TE	Negative	IVB	QT	FCEE-1.5M
11		43	TE/LP	NR	IVB	QT	FCEE-2M
12	Lowery *et al* [27] (USA 2009)	60	SPM	Inappropriate	IB1	RT + C	VSEE-120M
13	Balci *et al* [21] (Turquía 2010)	53	SPM	AdenoCA	IIIC1	C	NR
14	Banik and Dey [22] (India 2010)	43	SUA	AdenoCA	I	NR	NR
15	Yoon *et al* [23] (Corea 2011)	47	SPC	AdenoCA	IB1	C	FCEE-6M
16	Giordano *et al* [10] (Italia 2012)	45	SUA	NR	IIB	C	NR
17	Kaidar-Person *et al* [18] (Israel 2013)	37	SPC	NR	IIIC1	RT + C	VSEE-4M
18	Washimi *et al* [8] (Japón 2015)	31	SUA	Atypical	IIA	C + QT	VSEE-41M
19	Cracchiolo *et al* [24] (USA 2016)	64	DABD	AdenoCA	IVB	Palliative	FCEE-3M
20	Sal *et al* [7] (Turquía 2016)	48	SPC	NR	IB1	C	VSEE-18M
21	Doghri *et al* [31] (Tunes 2017)	48	SUA	NR	IVB	Palliative	FCEE-3M
22	Wang *et al* [9] (Taiwan 2018)	48	SPM/DABD	Negative	IVB	C + QT	VCEE-8M
23	Hamada *et al* [6] (Japón 2019)	40	SUA	NR	IB2	QT + Cx	VCEE-29M
24		44	NR	NR	IB1	C	VSEE-15M
25	Kawai *et al* [19] (Japón 2019)	40	ASINT	Atypical	IB1	C + QT	VSEE-33M
26	Kim *et al* [5] (Corea 2021)	43	SUA	Atypical	IIIC1	QTRT + QT	FCEE-15M
27	Li *et al* [20] (China 2021)	35	SPC	NR	IB2	C + QTRT	VSEE-12M
28	Salmen *et al* [15] (USA 2021)	50	SUA	Negative	IIA	QTRT + C	VSEE-12M
29	Pliego-Ochoa *et al* [16] (México 2021)	60	ASINT	LIE-AG	NR	C	VSEE-9M
30	Alexander-Rodríguez *et al* [11] (Colombia 2022)	31	SPC	LIE-BG	IIICI	C + QTRT	VSEE-12M
31	Purwoto *et al* [17] (Indonesia 2022)	39	SPC/DABD	NR	IB2	C + RT	VSEE-12M
32	Lazhar *et al* [4] (Marruecos 2023)	68	DPELV	NR	IVA	QTRT	FCEE-1M
33	Abad-Licham (Perú 2023)	51	SUA/DPEL	AdenoCA	IIIB	None	FCEE-1M

SUA: abnormal uterine bleeding, SPC: post coital bleeding, SPM: post menopause bleeding, DABD: abdominal pain, DPELV: pelvic pain, LP: lymphadenopathy, TE: thromboembolism, FV: vaginal discharge, ASINT: asymptomatic, AdenoCA: adenocarcinoma, QT: chemotherapy, QTRT: concurrent chemoradiotherapy, RT: radiotherapy, C: surgery, VSEE: alive without evidence of disease, VCEE: alive with evidence of disease, FCEE: deceased with evidence of disease, M: month, NR: not registered, PV: loss of sight

## References

[1] Sung H, Ferlay J, Siegel RL (2021). Global cancer statistics 2020: GLOBOCAN estimates of incidence and mortality worldwide for 36 cancers in 185 countries. CA Cancer J Clin.

[2] Gallardo-Alvarado L, Cantú-de León D, Ramirez-Morales R (2022). Tumor histology is an independent prognostic factor in locally advanced cervical carcinoma: a retrospective study. BMC Cancer.

[3] Rositch AF, Levinson K, Suneja G (2022). Epidemiology of cervical adenocarcinoma and squamous cell carcinoma among women living with human immunodeficiency virus compared with the general population in the United States. Clin Infect Dis.

[4] Lazhar H, Slaoui A, Rostoum S (2023). Primary signet ring cell carcinoma of the cervix: about an uncommon case report. Int J Surg Case Rep.

[5] Kim YH, Lee SJ, Lee SU (2021). Primary signet ring cell carcinoma of the uterine cervix: a case report. Medicine (Baltimore).

[6] Hamada K, Baba T, Takaori A (2019). Primary signet ring cell carcinoma of uterine cervix and related disease: two case reports and a review. Int Cancer Conf J.

[7] Sal V, Kahramanoglu I, Turan H (2016). Primary signet ring cell carcinoma of the cervix: a case report and review of the literature. Int J Surg Case Rep.

[8] Washimi K, Yokose T, Noguchi A (2015). Diagnosis of primary pure signet-ring cell carcinoma of the cervix. Pathol Int.

[9] Wang YC, Yu YL, Fan CW (2018). Primary signet ring cell carcinoma of the cervix: a case report with review of the literature. Taiwan J Obstet Gynecol.

[10] Giordano G, Pizzi S, Berretta R (2012). A new case of primary signet-ring cell carcinoma of the cervix with prominent endometrial and myometrial involvement: immunohistochemical and molecular studies and review of the literature. World J Surg Oncol.

[11] Alexander-Rodríguez J, Sánchez-Montoya M, Oliveros-Riveros L (2022). Primary signet ring cell carcinoma of the cervix: a case report and literature review. Ginecol Obstet Mex.

[12] Stolnicu S, Barsan I, Hoang L (2018). International Endocervical Adenocarcinoma Criteria and Classification (IECC): a new pathogenetic classification for invasive adenocarcinomas of the endocervix. Am J Surg Pathol.

[13] Stolnicu S, Barsan I, Hoang L (2018). Diagnostic algorithmic proposal based on comprehensive immunohistochemical evaluation of 297 invasive endocervical adenocarcinomas. Am J Surg Pathol.

[14] Stolnicu S, Hoang L, Chiu D (2019). Clinical outcomes of HPV-associated and unassociated endocervical adenocarcinomas categorized by the International Endocervical Adenocarcinoma Criteria and Classification (IECC). Am J Surg Pathol.

[15] Salmen N, LaBella D, Strumpf K (2021). A case of primary signet-ring cell cervical carcinoma treated with chemoradiation, brachytherapy, and adjuvant hysterectomy. Case Rep Obstet Gynecol.

[16] Pliego-Ochoa A, Cruz-Caramillo A, Martínez-Gómez H (2021). Primary signet-ring cell carcinoma of the cervix. A case report with review of the literature. Gac Mex Oncol.

[17] Purwoto G, Nuryanto KH, Wibowo TA (2022). Could combination of radical hysterectomy and radiation effective in the treatment of primary cervical signet ring cell carcinoma?: a rare case report. Int J Surg Case Rep.

[18] Kaidar-Person O, Amit A, Berniger A (2013). Primary signet-ring cell adenocarcinoma of the uterine cervix: case report and review of the literature. Eur J Gynaecol Oncol.

[19] Kawai S, Torii Y, Kukimoto I (2019). A case of primary signet-ring cell carcinoma of the cervix containing full genome of human papillomavirus. Indian J Pathol Microbiol.

[20] Li S, Gan F, Luo M (2021). A rare case of primary signet-ring cell cervical carcinoma: early stage with independent bilateral ovarian metastases. Oncol Targets Ther.

[21] Balci S, Saglam A, Usubutun A (2010). Primary signet-ring cell carcinoma of the cervix: case report and review of the literature. Int J Gynecol Pathol.

[22] Banik T, Dey P (2011). Signet ring cell carcinoma of the cervix on cervical smear. Diagn Cytopathol.

[23] Yoon A, Kim SH, Kim HJ (2011). Primary signet ring cell carcinoma of the uterine cervix: a case report. Korean J Obstet Gynecol.

[24] Cracchiolo B, Kuhn T, Heller D (2016). Primary signet ring cell adenocarcinoma of the uterine cervix – a rare neoplasm that raises the question of metastasis to the cervix. Gynecol Oncol Rep.

[25] Moritani S, Ichihara S, Kushima R (2004). Combined signet ring cell and glassy cell carcinoma of the uterine cervix arising in a young Japanese woman: a case report with immunohistochemical and histochemical analyses. Pathol Int.

[26] Veras E, Srodon M, Neijstrom E (2009). Metastatic HPV-related cervical adenocarcinomas presenting with thromboembolic events (Trousseau syndrome): clinicopathologic characteristics of 2 cases. Int J Gynecol Pathol.

[27] Lowery WJ, Difurio MJ, Sundborg MJ (2009). Cervical signet-ring cell carcinoma presenting as a synchronous primary carcinoma with uterine adenocarcinoma. Mil Med.

[28] Haswani P, Arseneau J, Ferenzcy A (1998). Primary signet ring cell carcinoma of the uterine cervix: a clinicopathologic study of two cases with review of the literature. Int J Gynecol Cancer.

[29] Suárez-Peñaranda JM, Abdulkader I, Barón-Duarte FJ (2007). Signet-ring cell carcinoma presenting in the uterine cervix: report of a primary and 2 metastatic cases. Int J Gynecol Pathol.

[30] Cardosi RJ, Reedy MB, Van Nagell JR (1999). Neuroendocrine signet ring cell adenocarcinoma of the endocervix. Int J Gynecol Cancer.

[31] Doghri R, Tounsi N, Slimane M (2017). A new case of primary signet ring cell carcinoma of the uterine cervix: a case report and review of the literature. J Cancer Sci Ther.

